# Enhanced degradation of pendimethalin by immobilized cells of *Bacillus lehensis* XJU

**DOI:** 10.1007/s13205-015-0299-0

**Published:** 2015-04-04

**Authors:** Veena S. More, Preeti N. Tallur, Francois N. Niyonzima, Sunil S. More

**Affiliations:** Department of Biochemistry, MSRCASC, Mattikere, MSRIT Post, Bangalore, 560054 India; Department of Chemistry, Government Arts and Science College, Karwar, 581301 India; Department of Maths, Science and PE, College of Education, University of Rwanda, Kigali, 5039 India; Department of Biochemistry, Center for Post Graduate Studies, Jain University, Bangalore, 560011 India

**Keywords:** Pendimethalin, *Bacillus lehensis* XJU, Biodegradation, Polyurethane foam, Immobilization

## Abstract

A bacterium capable of degrading pendimethalin was isolated from the contaminated soil samples and identified as *Bacillus lehensis* XJU based on 16S rRNA gene sequence analysis. 6-Aminopendimethalin and 3,4-dimethyl 2,6-dinitroaniline were identified as the metabolites of pendimethalin degradation by the bacterium. The biodegradation of pendimethalin by freely suspended and the immobilized cells of *B. lehensis* on various matrices namely agar, alginate, polyacrylamide, and polyurethane foam was also investigated. The batch degradation rate was nearly the same for both free and immobilized cells in agar and alginate, whereas polyacrylamide- and PUF-immobilized cells degraded 93 and 100 of 0.1 % pendimethalin after 96 and 72 h, respectively. At higher concentration, the degradation rate of freely suspended cells decreased; whereas the same immobilized cells on polyurethane foam completely degraded 0.2 % pendimethalin within 96 h. The repeated batch degradation with the polyurethane foam-immobilized cells was reused for 35 cycles without losing the 0.1 % pendimethalin degrading ability. In contrast, agar-, alginate- and polyacrylamide-immobilized cells could be reused for 15, 18, and 25 cycles, respectively. When the pendimethalin concentration was increased to 0.2 %, the immobilized cells could be reused but the pendimethalin degradation rate was decreased. Polyurethane foam-immobilized cells exhibited better tolerance to pH and temperature alterations than freely suspended cells and could be stored for more than 3 months without losing pendimethalin degrading ability. The immobilization of cells capable of degrading pendimethalin may serve as an ideal technique for the complete degradation of the herbicide in the environment.

## Introduction

Microorganisms are one of the tools used to detoxify toxic compounds present in the environment. Free suspended or immobilized microbial cells can be used for this purpose. However, the immobilized microbial cells have many advantages over free suspended cells under different conditions. For instance, the immobilization of whole cells increases degradation rate owing to increased cell population density, cell wall permeability, and extracellular microbial enzymes stability are improved, cells can be easily removed from the reaction mixture, higher operational stability and storage stability, reuse of immobilized cells in continuous reactors, and allows the bioreactors to operate at flow rates different from the growth rate of the microorganisms (Bettmann and Rehm 1984; Hall and Rao 1989; Cassidy et al. 1996; Ha et al. 2009; Zheng et al. 2009). In addition, the immobilized cell systems act as a protective cover in the presence of toxic compounds and are more resistant to pH or temperature changes. However, free suspended cells have better mass transfer aspects compared to immobilized bacterial or fungal cells (Trevors et al. 1992; Zheng et al. 2009).

In the last two decades, there have been intensive researches on the use of immobilized microbial cells as biocatalysts, using numerous reactors like fed batch, semi-continuous fed batch, and continuous packed bed reactor. Each reactor type possesses its disadvantages and advantages, and the choice of a particular type of a reactor may depend on the operational conditions, and inexpensive and non-toxic support inert material for microbial cell immobilization, etc., (Zheng et al. 2009). Bacterial cells immobilized on various matrices have been used extensively for biodegradation of various toxic nitroaromatics such as trinitrotoluene (TNT) (Rho et al. 2001; Ullah et al. 2010), nitrobenzene (Zheng et al. 2009; Qi et al. 2012), 2-nitrotoluene (Mulla et al. 2013), and 3-nitrobenzoate (Mulla et al. 2012).

Pendimethalin [*N*-(1-ethyl propyl) 2,6-dinitro-3,4-xylidine], a common water and soil contaminant, herbicide of dinitroaniline group, is used to control weeds in various crop plants. The use of pendimethalin may adversely affect endangered species of terrestrial and semi-aquatic plants and invertebrates (Kole et al. 1994). One of the best strategies to degrade the hazardous compounds (including pendimethalin) is to use microorganisms. There are few reports on the degradation of pendimethalin by free cells of *Fusarium oxysporum* and *Paecilomyces variotii* (Singh and Kulshrestha 1991), *Azotobacter chroococcum* (Kole et al. 1994), *Bacillus circulans* (Megadi et al. 2010), and fungus *Lecanicillium saksenae* (Pinto et al. 2012). However, there is no report on the degradation of pendimethalin by immobilized bacterial or fungal cells. The aim of the present investigation was therefore to compare the pendimethalin degradation by freely suspended and immobilized cells of *Bacillus lehensis* XJU on various matrices in batch and semi-continuous degradation, and to evaluate the effect of pH, temperature, and storage stability of pendimethalin degradation rate by polyurethane foam (PUF)-immobilized bacterial cells.

## Materials and methods

### Chemicals and reagents

Pendimethalin was a generous gift from Rallis Agrochemicals India Ltd. (Mumbai, India). The 6-aminopendimethalin and 3,4-dimethyl 2,6-dinitroaniline standards were purchased from Sigma Chemical Co. (St. Louis, USA). Polyurethane foam and nylon meshes were purchased from Merck Specialities Private Limited (Mumbai, India). Acrylamide, bisacrylamide, ammonium persulfate, sodium alginate (SA), nutrient agar, agar–agar, sodium chloride, and calcium chloride were purchased from HiMedia Laboratories (Mumbai, India). All other chemicals used were of analytical grade and available commercially.

### Isolation and identification of the bacterium

The bacterium was isolated from the contaminated soil samples by enrichment cultures with pendimethalin as a sole carbon source. It was identified based on morphological and biochemical aspects, as well as based on 16S rRNA gene sequence analysis. It was maintained on mineral salt (Seubert 1960) agar slants containing 0.1 % (w/v) pendimethalin.

### Isolation and identification of the metabolites of pendimethalin degradation by the isolate

The mineral salt medium (Seubert 1960) containing 0.1 % (w/v) pendimethalin was used as the production medium. The inoculated flasks were incubated on an orbital incubator (Model S150, Stuart, India) regulated at 150 rev/min for 24 h at room temperature (30 ± 2 °C). The bacterial growth was turbidometrically followed at 660 nm. The culture filtrate was collected by centrifugation, and ethyl acetate was used to extract the end products of pendimethalin degradation from the culture supernatant. The methanol was utilized to dissolve the residue from extraction. Thin layer chromatography (TLC) on silica gel G plates was used to analyze the residue for the presence of metabolites, using various solvent mixtures. An aqueous solution of FeCl_3_–K_3_Fe(CN)_6_ was used to visualize the metabolites after exposure to I_2_ vapors. The metabolites were also quantified by reversed-phase HPLC with a 5 µ spherisorb ODS (C_18_) column (250 × 4.6 mm). The mobile phase was a mixture of acetonitrile and 50 mM phosphate buffer of pH 7.0 in the (70: 30, v/v) ratio. The flow rate was 1 mL/min, and the peaks were detected at 254 nm. UV–visible spectrophotometer was used to record the metabolites absorbance spectra. The metabolites were also further subjected to mass spectral studies (JEOL DX303).

### Media used for the degradation studies

Two different media were used for this study. The mineral salt medium (MM1) consisting of (g/l) K_2_HPO_4_ (6.30), KH_2_PO_4_ (1.83), NH_4_NO_3_ (1.00), MgSO_4_· 7H_2_O (0.10), CaCl_2_·2H_2_O (0.10), FeSO_4_·7H_2_O (0.10), Na_2_MoO_4_·2H_2_O (0.005), and MnSO_4_· H_2_O (0.10) was used for the pre-cultivation of the bacterium. The medium was filtered and the pH was adjusted to 7.0. The medium was then dispersed in 100 mL quantities in 500 mL Erlenmeyer flasks and sterilized by autoclaving for 15 min at 15 psi. After sterilization by membrane filtration, pendimethalin was supplemented to the cultivation medium before bacterial inoculation. For the degradation studies, the mineral salt medium (MM2) comprising (g/l) K_2_HPO_4_ (6.30), NH_4_NO_3_(1.00), MgSO_4_·7H_2_O (0.20), CaCl_2_·2H_2_O (0.20), and FeCl_3_ (0.05) was used. The medium was filtered and the pH was adjusted to 7.0. The medium was then dispersed in 100 mL quantities in 500 mL Erlenmeyer flasks and sterilized by autoclaving for 15 min at 15 psi; 0.1/0.2 % (w/v) pendimethalin was then added after MM2 sterilization. The bacterial cultures were then incubated at 30 °C with shaking at 150 rpm. The plate count method was used to find out the bacterial cell concentration.

### Immobilization of bacterial cells in various matrices

*Bacillus lehensis* XJU was grown in the MM1 containing pendimethalin. During the mid-logarithmic growth, the bacterial cells were collected by centrifugation at 5000×*g* for 10 min at 15 °C, and washed two times with phosphate buffer (pH 7.0) of 50 mM strength. Alginate, agar, polyacrylamide (PA), and polyurethane foam (PUF) were then used as inert materials to immobilize the bacterial cells.

A procedure proposed by Bettmann and Rehm (1984) was used for sodium alginate entrapment of bacterial cells. 4 % (w/v) sodium alginate was mixed with 100 mL of distilled and autoclaved for 15 min. For biodegradation investigation, 20 g of wet bacterial cells was dissolved in 50 mL of MM2. The resulted suspension of *B. lehensis* XJU cells was mixed with 200 mL of alginate solution and stirred well with a magnetic stirrer. A combination of sodium alginate-bacterial cells was then added drop wise to a cold 0.2 M CaCl_2_ solution with a burette. The formed gel beds of about 2.5 mm diameter were hardened for 5 h by re-suspending into a freshly sterile solution of CaCl_2_ solution, and then frozen overnight at −18 °C. The beds were finally washed many times with sterilized distilled water and used for further studies.

The method proposed by Jonathan (1988) was followed for agar entrapment of bacterial cells. 4.5 mL of 0.9 % NaCl (w/v) was mixed with 100 mg of agar and heated at 100 °C till complete dissolution. The mixture was then cooled down at 40 °C. A slurry of *B. lehensis* XJU cells was taken and dissolved in 0.9 % (w/v) NaCl solution. 4.5 mL of the agar solution was mixed with 0.5 mL of bacterial cell slurry, poured on the nylon net placed on the glass plate, and cooled down to 5 °C. The 0.1 M phosphate buffer of pH 7.0 was used to store nylon membrane until used.

Jonathan (1988) method was also used for bacterial cells immobilization in polyacrylamide. 10 mL of distilled water was mixed with 15 g of wet bacterial cells and chilled in ice. 2.85 g acrylamide, 0.15 g bisacrylamide, and 10 mg ammonium persulfate were dissolved in chilled 10 mL of 0.2 M potassium phosphate buffer (pH 7.0). The bacterial cell suspension was immediately mixed with the buffer solution, poured into Petri plates, and allowed to polymerize for 1 h. 100 mL of 0.2 M potassium phosphate buffer (pH 7.0) was used to suspend the resulted sieved gels and allowed to settle down. After decantation, the gel was ready for degradation investigations.

Hall and Rao (1989) method was followed for polyurethane foam (PUF) immobilization. The PUF was cut into 5 mm cubes, washed with distilled water two times, and then dried. 4 g sterile foam cubes were placed in 100 mL of bacterial cell suspensions (9 × 10^9^ cfu/mL) contained in 500-mL Erlenmeyer flasks, mixed for 2 h with the help of magnetic stirrer, and shaken for 1 h at 150 rpm. The conical flasks were left undistributed for 2 more hours. After medium removal, a saline solution was used to wash the immobilized foam cubes for further studies.

### Pendimethalin degradation conditions

The batch degradations were carried out for both freely and immobilized *B. lehensis* XJU cells in four matrices. 3 mL of heat-killed cells along with 3 mL of freely suspended exponentially growing cells was added to 500 mL Erlenmeyer flasks containing 97 mL mineral salts medium (MM2) with different amount of substrates (0.1 and 0.2 %, w/v). For immobilized *B. lehensis* XJU cells, 11 g wet beads/foam cubes of the four matrices were supplemented to a 500 mL Erlenmeyer flasks containing 100 mL of mineral salts medium (MM2) with 0.1/0.2 % pendimethalin. The cell population in various matrices was in the 1.0 to 1.3 × 10^10^ cfu/g beads/foam cubes range. The degradation process by free and immobilized cells was carried out at room temperature (30 °C) on a rotary shaker (150 rpm). The various samples from culture medium were extracted at regular intervals for analysis of the residual substrate pendimethalin by high performance liquid chromatography (HPLC).

To evaluate the longevity of degrading activity of immobilized bacterial cells in various matrices, repetitive batch degradations were carried out. After each cycle of incubation period (96 h/cycle), the spent medium was decanted and beads/foam cubes were washed with double distilled water and transferred into a fresh MM2 medium containing pendimethalin. The degradation process was carried out under identical conditions and spent medium was used for the residual pendimethalin analysis by HPLC.

### Effect of pH and temperature on the pendimethalin degradation by freely suspended and PUF-immobilized cells

The rate of degradation of pendimethalin by freely suspended cells and PUF-immobilized cells of *B. lehensis* XJU at different pH (4.0–10.0) and temperatures (20–45 °C) was measured after 96 h of incubation.

### Storage stability of freely suspended and PUF-immobilized bacterial cells in degrading pendimethalin

The storage stability of both free suspended and PUF-immobilized cells was evaluated at 4 °C every 10 days for a period 90 days.

### Analytical methods

The pendimethalin in the spent medium was quantified by reversed-phase HPLC with a 5 µ spherisorb ODS (C_18_) column (250 × 4.6 mm). The mobile phase was a mixture of acetonitrile and 50 mM phosphate buffer of pH 7.0 in the (70: 30, v/v) ratio. The flow rate was 1 mL/min and the peaks were detected at 254 nm.

### Statistical analysis

Three independent experiments were conducted to evaluate pendimethalin degradation by freely suspended and immobilized bacterial cells. The means were compared by one-way analysis of variance (ANOVA) and means for groups in homogeneous subsets were given by Duncan’s multiple range test (DMRT) at the 5 % significance level. The SPSS statistical package (PASW Statistics 18) was utilized for all statistical evaluations.

## Results

### Biodegradation of pendimethalin by *Bacillus lehensis* XJU

The bacterium isolated from the contaminated soil samples by enrichment cultures with pendimethalin as a sole carbon source was identified as *B. lehensis* XJU based on 16S rRNA gene sequence analysis (GenBank Accession Number: AY793550). The analysis of metabolites of the culture filtrates of *B. lehensis* XJU grown on pendimethalin showed the presence of two compounds whose chromatographic and spectral values corresponded well with that of authentic 3,4-dimethyl 2,6-dinitroaniline and 6-aminopendimethalin (Table 1).

**Table 1:** Chromatographic and spectral properties of metabolites resulted from pendimethalin degradation by *Bacillus lehensis* XJU

Property	Isolated metabolite 1	Authentic 6-aminopendimethalin	Isolated metabolite 2	Authentic 3,4-dimethyl 2,6-dinitroaniline
TLC: *R*_f_ values in different solvent systems				
A: Hexane–ethyl acetate (1:1, v/v)	0.87	0.87	0.73	0.74
B: Toluene-dioxan-acetic acid (90:20:4, v/v)	0.93	0.94	0.83	0.83
UV absorption *λ*_max_ (nm)	229.43	229.43	234.42	234.42
HPLC retention time (min)	7.63	7.63	1.39	1.40
MS M^+^ at *m*/*z*	236, 220, 191	236, 220, 191	181, 121, 55	181, 121, 55

### Degradation of pendimethalin by freely suspended and immobilized cells of *Bacillus lehensis* XJU in batch cultures

**Fig. 1:**
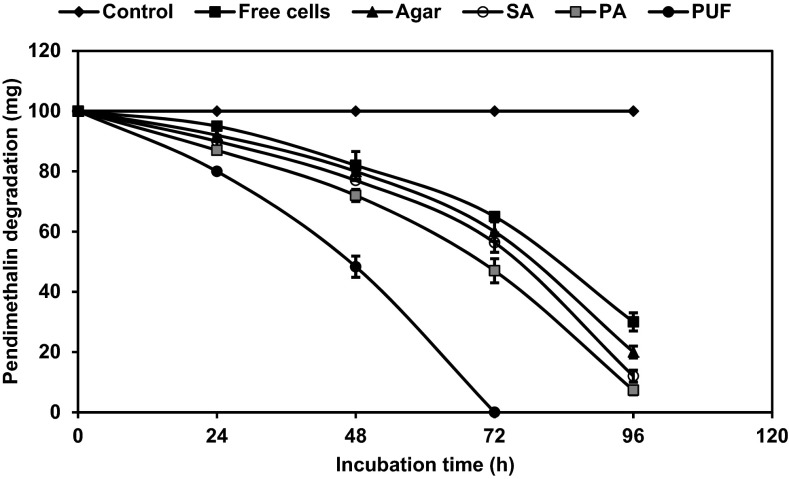
Batch culture degradation of 0.1 % pendimethalin by cells of *Bacillus lehensis* XJU immobilized on polyurethane foam (PUF, *filled circle*), polyacrylamide (PA, *filled square*), sodium alginate (SA, *open circle*), agar (*filled triangle*), and by free suspended cells (*filled square*). The uninoculated culture served as control (*filled diamond*)

**Fig. 2:**
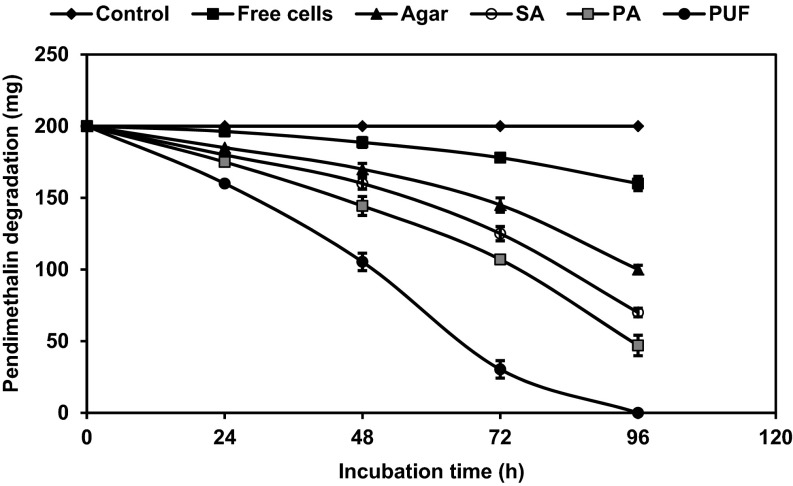
Batch culture degradation of 0.2 % pendimethalin by cells of *Bacillus lehensis* XJU immobilized on polyurethane foam (PUF, *filled circle*), polyacrylamide (PA, *filled square*), sodium alginate (SA, *open circle*), agar (*filled triangle*), and by free suspended cells (*filled square*). The uninoculated culture served as control (*filled diamond*)

Batch degradation on shaken cultures with *B. lehensis* XJU showed that increasing concentration of pendimethalin was better tolerated and quickly degraded by immobilized cells than by free organisms. At low concentration (0.1 % w/v), the degradation rate was nearly the same for both free and immobilized cells in agar, alginate, and polyacrylamide after 96 h, with complete degradation in bacterial cells immobilized in PUF after 72 h (Fig. 1). However, with increasing concentration (0.2 % w/v), the degradation rate of free cells decreased and 20 % pendimethalin was only degraded; whereas the same immobilized cells in agar, alginate, and polyacrylamide matrices degraded 50–77 %. A complete degradation of pendimethalin was also observed for cells immobilized in PUF but within 96 h (Fig. 2). At the concentration of 0.7 % w/v, pendimethalin was not degraded by free cells, but was degraded by PUF-immobilized cells (data not shown), which were more effective than cells immobilized in agar, alginate, and polyacrylamide.

### Semi-continuous degradation of pendimethalin by immobilized cells of *B. lehensis* XJU

**Fig. 3:**
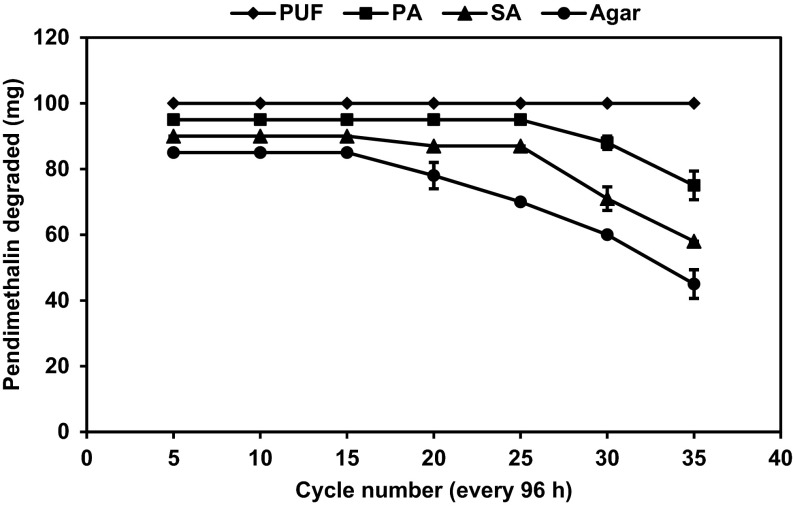
Semi-continuous degradation of pendimethalin (0.1 %) by cells of *Bacillus lehensis* XJU immobilized on polyurethane foam (PUF, *filled diamond*), polyacrylamide (PA, *filled square*), sodium alginate (SA, *filled triangle*), and agar (*filled circle*)

**Fig. 4:**
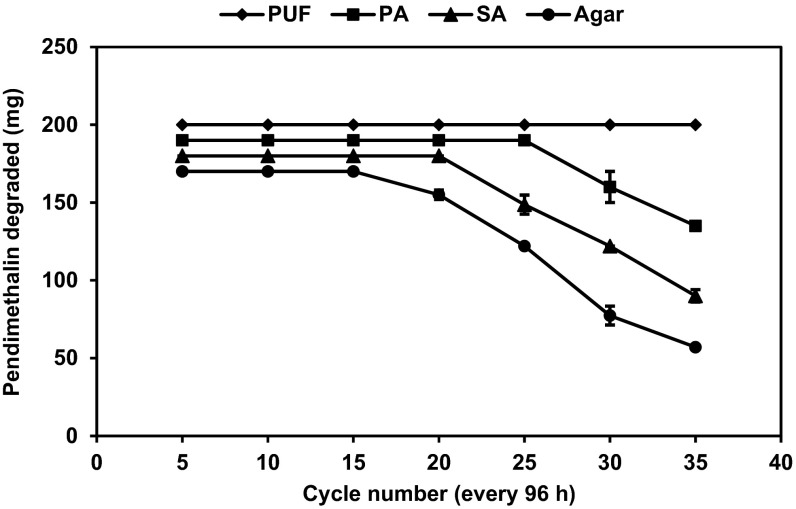
Semi-continuous degradation of pendimethalin (0.2 %) by cells of *B. lehensis* XJU immobilized on polyurethane foam (PUF, *filled diamond*), polyacrylamide (PA, *filled square*), sodium alginate (SA, *filled triangle*), and agar (*filled circle*)

The degradation of pendimethalin by cells immobilized in polyurethane foam (PUF), alginate, agar, and polyacrylamide was carried out at different concentrations of pendimethalin (0.1 and 0.2 %) for 96 h. The polyurethane foam (PUF)-immobilized cells were reused for 35 cycles without losing the pendimethalin degrading capacity when the initial concentration of pendimethalin was 0.1 %. In contrast, agar-, alginate-, and polyacrylamide-immobilized cells were reused for 15, 18, and 25 cycles, respectively, (Fig. 3). When the initial concentration of pendimethalin was increased to 0.2 %, the immobilized cells could be reused but the rate of degradation of pendimethalin was decreased (Fig. 4).

### Effect of pH on pendimethalin degradation rate by PUF-immobilized bacterial cells

**Fig. 5:**
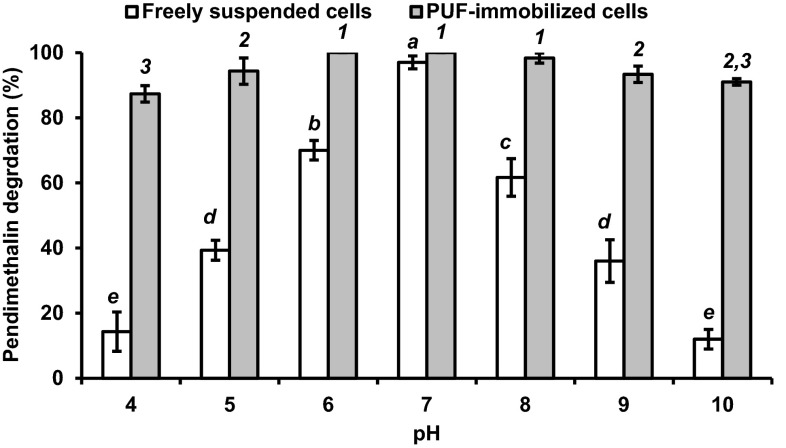
Effect of pH on the degradation of 0.1 % pendimethalin by freely suspended (*open square*) and PUF-immobilized (*filled square*) *Bacillus lehensis* XJU cells. The pendimethalin degradation values with different numbers or alphabets on the *error bars* significantly differ from each other at *P*_0.05_

The effect of pH on degradation rates of pendimethalin by immobilized cells on PUF and free suspended cells was investigated in the pH 4.0–10.0 range. The initial pH alteration in the 6.0 and 8.0 range had no effect on the pendimethalin degradation by PUF-immobilized cells. A slight degradation rate was observed at pH 4.0, 9.0, and 10.0. However, freely suspended cells were active at pH 7.0, and other pH below or above 7.0 had adverse effects on both degradation rate and organism growth (Fig. 5).

### Effect of temperature on pendimethalin degradation rate by PUF-immobilized bacterial cells

**Fig. 6:**
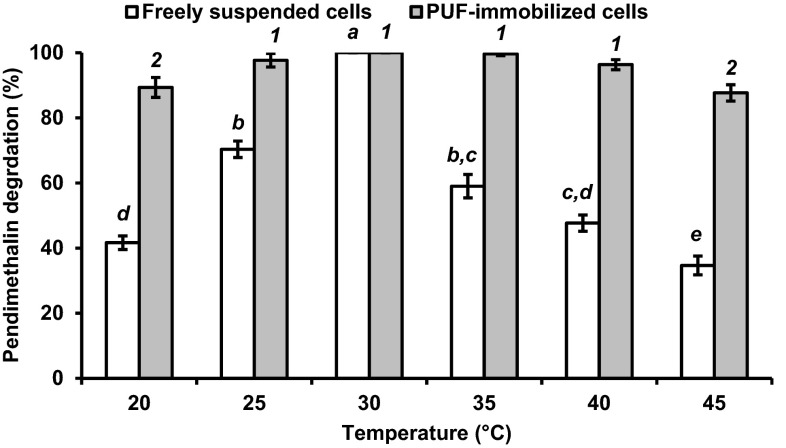
Influence of temperature on the 0.1 % pendimethalin degradation by freely suspended cells (*open square*) and PUF-immobilized *Bacillus lehensis* XJU cells (*filled square*). The pendimethalin degradation values with different numbers or alphabets on the *error bars* significantly differ from each other at *P*_0.05_

The effect of temperature on the degradation of pendimethalin by PUF-immobilized cells was analyzed. The pendimethalin degradation was seen in the 20–45 °C range with optimum at 30 °C, although statistically at par with 25, 35, and 40 °C for immobilized bacterial cells. However, the temperatures below and above 30 °C were not suitable for degradation of pendimethalin by freely suspended cells (Fig. 6).

### Storage stability of the PUF-immobilized cells degrading pendimethalin

**Fig. 7:**
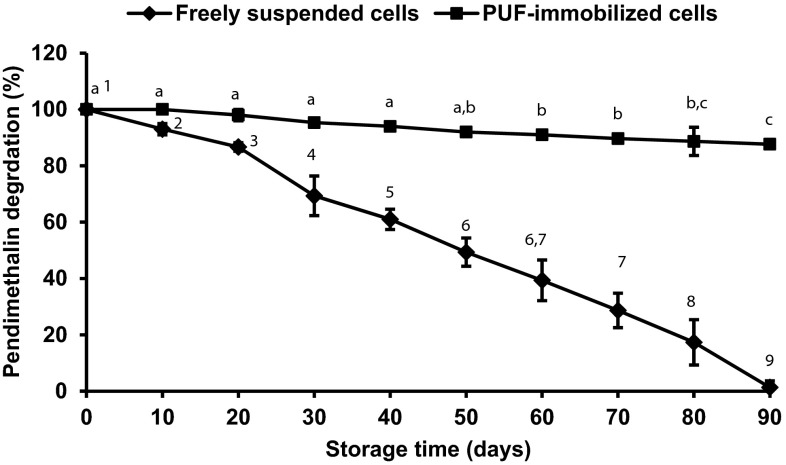
Storage stability of freely suspended (*filled diamond*) and PUF-immobilized (*filled square*) *Bacillus lehensis* XJU cells grown on 0.1 % pendimethalin. The pendimethalin degradation values with different numbers or alphabets on the *error bars* significantly differ from each other at *P*_0.05_

PUF-immobilized cells were stored at 4 °C for 90 days and no decline in degradation ability observed for 50 days, and a marginal decrease of less than 10 % was observed after 90 days. However, a significant gradual decrease in pendimethalin degradation ability was seen with freely suspended bacterial cells and no degradation observed after 3 months (Fig. 7).

## Discussion

The degradation of pendimethalin by *B. lehensis* XJU based on TLC, reverse phase HPLC, UV visible, and mass spectral studies has resulted in the formation of 3,4-dimethyl 2,6-dinitroaniline and 6-aminopendimethalin. Similarly, the biodegradation of the herbicide pendimethalin by *B. circulans*, resulted in the formation of two metabolites, viz., 6-aminopendimethalin by pendimethalin reduction, and 3,4-dimethyl 2,6-dinitroaniline by pendimethalin oxidative dealkylation (Megadi et al. 2010). Biodegradation of pendimethalin by freely suspended cells of *B. lehensis* XJU and by cells immobilized in agar, SA, PUF, and polyacrylamide was compared with respect to their degradation rate and tolerance against increasing concentrations of pendimethalin. In batch cultures, the freely suspended cells degraded pendimethalin comparatively well at lower concentrations (0.1 %) and degradation rate decreased at higher concentrations. The cells immobilized in SA, PUF, agar, and polyacrylamide matrices were able to survive and degrade pendimethalin at higher concentrations. The cells immobilized in PUF degraded pendimethalin up to the concentration of 0.7 % (w/v) in batch cultures. The present results revealed that the toxicity of pendimethalin at high concentration level could inhibit the metabolism resulting in lower removal efficiency by the free cells. In addition, it indicates that in an immobilized cell culture, the carrier material act as a protective cover against the toxicity of pendimethalin. The enhanced pendimethalin degradation rate can also be ascribed to higher cell population density and higher activity of the cells immobilized in or on these carrier inert materials. Similarly, the inert materials served as a protective cover towards 2-nitrotoluene (Mulla et al. 2013) and 3-nitrobenzoate (Mulla et al. 2012). Likewise, the higher degradation rates of various toxic nitroaromatics were attributed to higher cell density in the matrices (Cassidy et al. 1996; Qi et al. 2012). Increased concentration of pendimethalin was better tolerated and degraded by PUF-immobilized cells than *B. lehensis* XJU cells immobilized in agar, SA, and polyacrylamide, as well as free cells. The cells of Micrococcus *luteus* Z3 immobilized in PUF had the higher nitrobenzene degradation capacity compared to free suspended cells (Qi et al. 2012). The moderate degradation rate observed with bacterial cells immobilized in agar, alginate, and polyacrylamide can be attributed to the leakage that may result from the mechanical instability and monomer and/or radical toxicity (Hall and Rao 1989; Trevors et al. 1992).

The semi-continuous degradation data showed that polyacrylamide and PUF-immobilized cells retained the pendimethalin degrading ability for a longer period since they could be reused for 26 and 35 cycles, respectively. But when the concentration of pendimethalin increased, polyurethane foam (PUF)-immobilized cells degraded pendimethalin faster than polyacrylamide-immobilized cells, which suggest that the PUF immobilization is a better technique for the degradation of toxic herbicides in the environment. However, the bacterial cells immobilized in SA and agar exhibited lower degradation ability of pendimethalin with increased cycle numbers. This was ascribed to the mechanical instability and gradual cell leakage from the inert porous beads (Trevors et al. 1992; Ha et al. 2009). Similarly, polyurethane foam was also an excellent support for the degradation of numerous nitroaromatics by bacterial cells (Zheng et al. 2009; Mulla et al. 2012, 2013). The increased degradation rate was ascribed to adsorbing capacity, stability, mechanical strength, and high porosity for the PUF-immobilized cells (Romaškevic et al. 2006).

The immobilized cells on PUF exhibited an excellent resistance to pH and temperature alterations, and had the better storage stability of more than 3 months than freely suspended cells in degrading pendimethalin. Similarly, the free and PUF-immobilized *Micrococcus* cells were active in the pH 7.0–8.0 at 30–35 °C and 5.0–10.0 at 25–40 °C, respectively, in degrading 2-nitrotoluene (Mulla et al. 2013). Likewise, a narrow range of pH (6.5–7.5) and temperature (30–35 °C), and a broad range of pH (5.0 to 10.0) and temperature (20–40 °C) were observed for freely suspended and PUF-immobilized cells, respectively, in degrading 3-nitrobenzoate (Mulla et al. 2012). The immobilized cells could be stored more than 3 months without losing too much pendimethalin degradation capacity. Similarly, the PUF-immobilized cells of *Bacillus flexus* strain XJU-4 and *Micrococcus* sp. strain SMN-1 were stored for 60 and 70 days at 4 °C without losing the capacity to degrade 3-nitrobenzoate (Mulla et al. 2012) and 2-nitrotoluene (Mulla et al. 2013), respectively. An effective biodegradation of elevated concentration of dinitroaniline herbicides (such as pendimethalin) and various nitroaromatic compounds can be achieved using immobilized microbial technology.

## Conclusion

The present investigation has revealed the biodegradation of pendimethalin by the bacterial isolate *B. lehensis* XJU. It also showed the pendimethalin biodegradation by freely suspended and immobilized *B. lehensis* XJU on different matrices. The microorganisms capable of degrading toxic compounds can therefore be immobilized by entrapment in an inexpensive supports and the immobilized cells retain their ability over a considerable period of time, especially for PUF. Thus, the immobilized microbial cell systems may find applications in the treatment of various contaminated environment sites. However, prior to large-scale application of such systems, further studies are needed for determining the optimal operating conditions.
